# Addictive Motivational Scaffolds and the Structure of Social Media

**DOI:** 10.1007/s11229-025-05312-z

**Published:** 2025-10-22

**Authors:** Lorenzo Manuali

**Affiliations:** University of Michigan, Department of Philosophy, Ann Arbor, MI, United States of America

**Keywords:** Addiction, Social Media, Motivational Scaffolding, Mental Scaffolding, Psychiatric Externalism, 4E Cognition

## Abstract

In this paper, I propose an account of behavioral addiction in terms of what I call addictive motivational scaffolds (AMSs). Taking inspiration from recent work concerning psychiatric externalism and addiction, I propose and describe the concept of motivational scaffolding: external structure that enhances, supports, or regulates motivational processes in the mind-brain. I then argue that some motivational scaffolds are likely difference-makers in that they make an activity more addictive. The paper proceeds in three main parts. First, I describe the concept of a motivational scaffold and how it builds on recent literature in 4E cognition/psychiatric externalist accounts of addiction. Using gambling and gaming as paradigm cases of addictive activities, I then identify and empirically justify four addictive motivational scaffolds (AMSs): (1) quantified metrics, (2) reward uncertainty, (3) short time-horizon to reward, and (4) physically salient features. Finally, I apply my account to social media to showcase its philosophical usefulness: analyzing behavioral addiction in terms of AMSs uniquely elucidates the more *structural* aspects of the addictiveness of social media, which are undertheorized.

There are growing literatures on why phenomena like social media (Carbonell & Panova, 2017; Sun & Zhang, 2021), pornography (de Alarcón et al., 2019; Duffy et al., 2016), and digital products generally are addictive and whether they ought to be considered (behavioral) addictions (Almourad et al., 2020; Lembke, 2021b; Waters, 2021).^1^ Most prominently, there is significant public debate concerning whether to restrict access to or otherwise regulate social media partly in virtue of its perceived addictiveness (Stokel-Walker, 2024). Australia, for instance, recently banned social media use for under-16s (Ritchie, 2024).

What makes some behaviors addictive?^2^ And, in particular, what makes these newer forms of digital behavior more addictive than other activities (e.g., swimming)? There are several levels of analysis at which this question can be answered. The Research Domain Criteria (RDoC) contains classic levels used in the sciences: physiology, neural circuitry, genes, and more (National Institute of Mental Health, n.d.).

There’s also now a fledgling literature concerning *psychiatric externalist* accounts of addiction. This framework “focuses on the empirical and conceptual centrality of the wider extra-neural environment to cognitive and mental processes” (Glackin et al., 2021, p. 1). As an example of such an explanation, both Glackin et al. (2021) as well as Lavallee and Osler (2024) provide useful analyses of how *affordances* shape addiction. This approach sheds new theoretical light on how addiction affects agency and provides practical fruits for approaches to treatment and recovery.^3^

With respect to psychiatric externalist accounts focused on *behavioral* addiction(s), many have fruitfully drawn on the concept of *mental scaffolding* (Timms & Spurrett, 2023; Voinea et al., 2024). Though there are various kinds of scaffolding, I intend to more fully develop the concept of *motivational scaffolding*, defined as external structure that changes the way motivational processes operate and are regulated in the mind-brain.

There are various ways in which motivational scaffolding is instantiated. Some motivational scaffolds are more addictive than others. Call more addictive motivational scaffolds *addictive motivational scaffolds (AMSs)*.

Addictive motivational scaffolds (AMSs), I argue, can provide us with a compelling explanation as to why certain behaviors (like gambling and gaming) are addictive. In the process, we end up with an interesting and novel account of behavioral addiction.

In my account, I identify four addictive motivational scaffolds: (1) Quantified Metrics, (2) Reward Uncertainty, (3) Short Time-Horizon to Reward, and (4) Physically Salient Features. I show how each AMS is present in (behaviorally) addictive activities (gambling and gaming in particular) and use empirical evidence from a variety of fields (e.g., neuroscience, gambling studies, and STS) to give a causal story as to why each is addictive.^4^ These two pieces of evidence ground the claim that each AMS leads to an activity becoming more addictive.

To be clear, I do not mean to make overly strong claims about the relationship between AMSs and behavioral addiction. I do not claim that the AMSs are necessary for behavioral addiction. Nor do I mean to suggest that they are jointly sufficient as a set. Nor do I even mean to suggest that any one of the AMSs is necessarily a difference maker – or that AMSs are necessarily a difference maker when considered as a set.

Rather, the strongest claims I want to make are the following: Keeping in mind that the extent to which an activity is (behaviorally) addictive is graded/dimensional, if an activity instantiates these four AMSs, then it is highly likely that it will be more behaviorally addictive to a significant degree. And so, an activity that instantiates all the AMSs to a substantial degree is highly likely to be addictive. Furthermore, each individual AMS is likely to be a difference maker in that, if it is instantiated, it is likely to make an activity more addictive that it otherwise would have been.

It’s also important to note that each AMS is graded/dimensional, and so one activity that possesses one AMS in abundance might be more addictive on the whole than another with a more moderate amount of multiple AMSs.

One of the main fruits of this account is that it offers a novel explanation of certain structural aspects of an activity that is attracting attention in the behavioral addiction literature: social media use. There is a small but growing literature on the role of various kinds of mental scaffolding (especially affective scaffolding) at play in our interaction with social media (Krueger & Osler, 2019; Steinert et al., 2022). But this literature doesn’t explicitly discuss the role of *motivational* scaffolding in social media. And even where (addictive) motivational scaffolding is implicitly discussed, discussion centers around social media content, social rewards, and algorithmic recommendation systems (Narayanan, 2022; Voinea et al., 2024). This picture misses a lot, and I use my account of addictive motivational scaffolds (AMSs) to offer a more *structural* account of the addictive nature of social media by identifying AMSs in social media platforms.

Before proceeding, a caveat: I intend for this account to elucidate some of the main ways motivational scaffolding can shape behavioral addictions. I have certainly not captured every way in which motivational scaffolding affects behavioral addiction – though I do think I’ve captured some of the main ways. Especially since there’s good reason to think that addiction is a heterogeneous phenomenon than defies uniform explanation (Epstein, 2020; Pickard, 2022, 2024), we shouldn’t expect any one account to capture every aspect of behavioral addiction.

## What is Motivational Scaffolding? Why Does it Matter?

Before delving into the AMSs, it’s important to clear some conceptual ground concerning motivational scaffolding and explain the virtues of the account of behavioral addiction on offer in the process.

Discussed widely in 4E cognition, mental scaffolding is any external structure that changes the way a given mental process operates or is regulated (Saarinen, 2020; Sterelny, 2010).^5^ External means “to a significant extent beyond the ‘skull or skin’” (Timms & Spurrett, 2023, p. 56). And structure is meant to be conceived of broadly to include “objects, mechanisms, temporally organised processes, symbolic systems, public language, and the activity of other agents” (Timms & Spurrett, 2023, p. 56).

Scaffolding can come in many forms. *Cognitive* scaffolding is mental scaffolding that “changes the cognitive demands of a task” (Timms & Spurrett, 2023, pp. 55–56). Think of how an agenda book can support the cognitive process of memory by reducing the cognitive demands of remembering your schedule. *Affective* scaffolding is a bit trickier to define, but it roughly refers to when an external structure changes how our affective states are enabled, supported, or regulated (Colombetti & Krueger, 2015; Saarinen, 2020, p. 820). Think of how consuming coffee supports a particular set of feelings throughout your day (and how its lack can lead to a set of undesirable feelings). *Attentional* scaffolding is mental scaffolding that “systematically influence users’ focus, management, and direction of attention, either benefitting or harming their ability to achieve their goals” (Voinea et al., 2024, p. 686).

Crucially for our purposes, ***motivational* scaffolding is external structure that changes the way motivational processes operate or are regulated in the mind-brain.**

As an example of motivational scaffolding, consider the layout of a supermarket. Sweets are placed towards the checkout because it takes less motivation to merely grab a sweet towards the end of your shopping trip – as opposed to actively seeking out sweets during the shopping trip.^6^ Consider also the hierarchical structure of a corporation or the incentive structure of a given worker’s salary – both change the way motivational processes operate and are regulated by employees (and probably managers).^7^

Here, one might have an objection: Motivational scaffolding seems rather thin. The worry is that too many things can count as motivational scaffolding, and so any unification of multiple scaffolds under “motivational scaffolding” is a cheap theoretical unification. But this would be too quick. While it’s true that lots of things can count as motivational scaffolding, they all work on a relatively limited set of neural and computational processes: the mind-brain’s internal reward system. By “the mind-brain’s internal reward system,” I mean the system theorized in psychology, cognitive science, and neuroscience that paradigmatically deals with the representation, perception, and processing of reward and connecting such perception and processing to action via motivation. In neuroscience, this system primarily corresponds to a number of interconnecting, physically instantiated neural circuits, perhaps the most prominent being the mesolimbic dopamine pathway/system (Lewis et al., 2021; Schultz, 2006). And so various mechanisms of motivational scaffolding are unified with respect to something rather specific – how and where they ultimately operate in the mind-brain.^8^

In discussions of mental scaffolding, motivational scaffolding has only been mentioned in passing, and it’s received scant (if any) theoretical attention (Colombetti & Krueger, 2015; Timms & Spurrett, 2023). This might be (at least in part) because some would say that it’s a type of affective scaffolding – either for more explicit reasons or to maintain a pluralistic conception of affect (Saarinen, 2020; Spurrett, 2024).^9^ But whether motivational scaffolding is a kind of affective scaffolding does not matter for our purposes.

What does matter is that some motivational scaffolding makes motivational processes more consistent with those in addiction. Put roughly: Some kinds of motivational scaffolding are more addictive than others.^10^ For instance, the kinds of motivational scaffolding instantiated in a video slot machine are more addictive than those instantiated in the layout of a supermarket (or even the lottery).

So it’s surprising that psychiatric externalist accounts of addiction, despite invoking mental scaffolding (e.g., cognitive scaffolding) haven’t previously made much use of motivational scaffolding (Glackin et al., 2021; Lavallee & Osler, 2024; Spurrett, 2024; Timms & Spurrett, 2023). Timms and Spurrett offer some analysis of the addictive nature of electronic gambling in terms of motivational scaffolding – or, at the very least, mental scaffolding that is motivation-adjacent (Spurrett, 2024; Timms & Spurrett, 2023). Colombetti and Krueger (2015) do as well. But none of these authors focus on motivational scaffolding primarily, and there are several addictive motivational scaffolds (AMSs) I discuss that they do not.

My own account of AMSs in behavioral addiction fills this gap in the literature by proposing several novel ways in which motivational scaffolding can make a given activity addictive. A number of the AMSs I discuss have been discussed elsewhere. But by elucidating more specifically how these external structures bear causal relationships to motivational processes in the mind-brain, I offer a new way of unifying them in the theoretical framework of (addictive) motivational scaffolding.^11^

One upshot of this account is that we can shed new theoretical light on the addictiveness of social media. By analyzing social media in terms of addictive motivational scaffolds, we elucidate new or underdiscussed pathways in which the external structure of social media influences our motivational processes so as to make engaging with it more addictive.

In particular, AMSs offer us more insight into the *structural* aspects of social media that make it addictive. The literature concerning social media addiction tends to focus on content, personalization, social rewards, and algorithmic recommendation systems (Carbonell & Panova, 2017; Narayanan, 2022; Sun & Zhang, 2021; Voinea et al., 2024). Where it does discuss structural aspects of social media (Flayelle et al., 2023, p. 140; Montag et al., 2019; Narayanan, 2022), it has missed or sidelined several ways in which the structure of social media platforms can facilitate addiction. Using the AMSs from my account, we also end up with a novel contribution to the literature on social media and addiction.

For each addictive motivational scaffold (AMS), I’ll first discuss how it appears in two cases of activities susceptible to behavioral addiction: gambling and gaming. Since both are more addictive activities (than, e.g., swimming), this gives us presumptive evidence that the motivational scaffold in question makes the activity in which it is instantiated more addictive.^12^ To give even more evidence that the given motivational scaffold is addictive, I’ll then give a causal story that explains its relationship to addictive behaviors.

## Addictive Motivational Scaffolds (AMSs)

To justify that AMSs lead to (behavioral) addictiveness, besides analyzing empirical evidence, I will focus on how they manifest in two paradigm cases of behavioral addiction: gambling and gaming. Before I can do that, however, I need to establish gambling and gaming as paradigm cases of behavioral addiction. And before I can do that, I need to give at least a bare bones account of what I mean by ‘addiction’.

So, here’s a working definition of addiction that focuses more on its behavioral outputs: Addiction is a dimensional phenomenon related to use of a given substance or taking part in a particular activity in which at least some of the following signs and symptoms occur:^13^

- Compulsive seeking of the activity or substance in question
- Persistent craving for the activity or substance in question
- Excessive risk-taking, especially related to the substance or activity in question
- Continued engagement with the substance or activity in question despite negative consequences
- Wanting or intending to cut down on engagement with the substance or activity, while failing to do so (or finding it excessively difficult to do so)
- Inability to complete other goal-directed tasks
- Neglect of responsibilities, relationships, or activities one used to care about

As for paradigmatic cases of behavioral addiction: I focus on gambling and gaming.

Gambling addiction is perhaps the most prominent example of behavioral addiction in the psychological and neurobiological literatures (American Psychological Association, 2013, pp. 585–589; Petry et al., 2018; Schüll, 2012, p. 17).

Certain forms of video gaming also constitute a classic case of a (behaviorally) addictive activity. Some neuroscientists recognize video games as an activity with clear addictive potential (Lembke, 2021a, 2021b; Petry et al., 2018). Internet Gaming Disorder is currently recognized in the DSM-5 as a “Condition for Further Study”, with a prototypical list of signs and symptoms that are substantially similar to gambling addiction’s (American Psychological Association, 2013, pp. 795–798).

Crucially, not all forms of gambling and gaming are equally addictive. When it comes to gambling, as Schüll writes, “most researchers place different forms of gambling along a continuum of intensity that progresses from lottery, bingo, and mechanical slots to sports, dice, cards, and finally, to video slots and video poker” (Calado & Griffiths, 2016, pp. 609–610; Schüll, 2012, p. 18). As for gaming, MMOs (massive multiplayer online games — think World of Warcraft), and especially MMORPGs (‘RPG’ being role-playing games — games in which you take on a particular character and play a specific role) are far more addictive than other games.

We’ve established gambling and gaming as paradigm cases of addiction. I’ll now identify the AMSs and justify that they are likely difference-makers with respect to addictiveness by arguing that they appear in these paradigmatic cases of behavioral addiction and by analyzing empirical evidence from a variety of fields.

## Quantified Metrics

I’ll argue that *quantified metrics* are an addictive motivational scaffold (AMS) by showing how they appear in gambling and more addictive forms of gaming. These metrics usually somehow delineate success in the task at hand.

Some activities contain more quantified metrics than others. For instance, some games revolve more around quantified metrics (e.g., experience points) whereas others do not (e.g., “good” or “evil” endings).

We find quantified metrics in one of the most paradigmatic addictive activities: gambling. The next win on the craps table, the next payout on the slot machine — there’s usually *one* denomination of reward and *one* way to win it (usually, chips or credits, which can be exchanged for cash). And these rewards are all quantifiable – they all come in numeric denominations and can be exchanged for cash.

We also find quantified metrics in more addictive games. The literature on *gamification* can be useful here. Having “immediate, vivid, and quantified evaluations” of success makes us crave the achievement of such scores (Browning & Adams, 2023, p. 5; Nguyen, 2021, p. 411). We want to “watch those numbers go up and up” (Nguyen, 2021, p. 411). MMORPGs – the most addictive kind of game – contain quantified metrics of some kind without fail (e.g., experience points, in-game currency).

But, of course, many kinds of game have quantified metrics. So, to show that quantified metrics are an AMS, I’ll also point to a type of game that is strongly motivating and in which quantified metrics play an outsized role: idle games. Idle games are a type of game in which the main objective is usually to increase one or a set of quantified metrics (e.g., in Cookie Clicker, the goal is to increase the number of cookies). Much of the game runs automatically, with the user checking on it and making adjustments. The user might make plans for how to increase their score further and make adjustments on that basis (Alharthi et al., 2018; Cutting et al., 2019; Spiel et al., 2019). Such games are very popular – a clear sign that they are strongly motivating. This does not necessarily make the games themselves very addictive – though, since addiction is a dimensional phenomenon, it would make them more addictive than other activities (like, e.g., swimming). But, because quantified metrics play such a crucial role in these strongly motivating games, idle games do provide us evidence that quantified metrics are an AMS.

In a similar vein, one might have an objection: Lotteries have a quantified metric (namely, money), and yet they’re not as addictive as other games/gambling (Calado & Griffiths, 2016, pp. 609–610; Schüll, 2012, p. 18). I have two responses. First, lotteries are still addictive (even if they are less addictive than other games/gambling). But second, even if that weren’t the case, there will conceivably be activities that possess, for example, one of the AMSs but are not very addictive activities. That’s okay. Possessing one AMS need not immediately imply that the activity is extremely addictive. In this case, lotteries might have quantified metrics and they might be somewhat addictive, but they lack other AMSs, such as short time-horizon to reward and physically salient features.

Why is it that quantified metrics are an addictive motivational scaffold? Quantified metrics exploit an attentional bias we have towards simpler information structures in order to keep us attended, engaged, and motivated to continue the activity in question.^14^

When we learn, our attention is biased towards simpler information structures – probably to reduce cognitive load (Galdo et al., 2022). By “simplicity”, Galdo et al. (2022) roughly refer to information structures that have fewer *dimensions*. To be more precise, Galdo et al. (2022) state that “simplicity” is characterized by representations that have a smaller number of stimulus dimensions (Galdo et al., 2022). Stimulus dimensions, as I read them, are the properties of a given stimulus that are relevant for perception or cognition (e.g., how thick a given line is, how thin a given line is, etc.) (Ashby & Maddox, 2005, pp. 152–153; Galdo et al., 2022).

In STS, the portability theory of information sheds light on what happens when context-rich, nuanced information gets quantified. Namely, there’s a “flattening” of the information in which context-sensitivity and nuance are stripped away (Merry, 2016; Nguyen, 2021; Porter, 1996; Scott, 1998). In other words, when put in terms of quantified metrics, complex phenomena are necessarily simplified. They are also almost certainly simplified in the sense specified by Galdo et al. (2022): there are fewer properties relevant for cognition or perception, since there are fewer properties *simpliciter*. And so, information structures with quantified metrics will be “simpler” in Galdo et al.’s (2022) sense.

Now, observe that attentional bias toward addiction-related “cues plays a role in development and maintenance of addictive behaviors” (Field et al., 2014; A. Jones et al., 2021, p. 413; Robbins & Ehrman, 2004).^15^ It’s also common ground that various kinds of learning play important roles in the development and maintenance of addiction – whether directly or indirectly (Berridge, 2007; Hyman, 2005; Ngetich et al., 2024).^16^

So, (1) attentional biases help develop and maintain an addiction, and (2) learning is also crucial to the development and maintenance of an addiction. Given these facts and that (3) we have an attentional bias towards simple (and thus quantified) information structures in learning, quantified metrics are plausibly a more addictive motivational scaffold.

Why? Presumably, (3) gives us reason to believe that we have an attentional bias towards simple information architectures in learning – and thus towards quantified metrics. If both learning and attentional biases are crucial aspects of addiction (per (1) and (2)), that inserts quantified metrics into two cognitive processes that are central to the development and maintenance of an addiction. While it would be empirically premature to say any more, this observation provides a plausible, high-level explanation of why quantified metrics constitute a motivational scaffold that is addictive (i.e., an AMS).

## Reward Uncertainty

Let’s move on to another AMS. An activity has more *reward uncertainty* (1) to the extent that whether the reward(s) will instantiate themselves is uncertain and (2) to the extent that the size of the reward(s) is uncertain.

There’s an important distinction to be made here: I’m not claiming that uncertainty *simpliciter* is an AMS, but only *reward* uncertainty.

To claim that uncertainty *simpliciter* is an AMS would, I think, be unwise. Why? Because certain kinds of reliability plausibly play an important role in at least some addictions (Lavallee & Osler, 2024; Ross, 2020). In behavioral addiction, the importance of reliability might manifest in the fact that, for example, gambling reliably produces “the zone” – a kind of flow state characterized by safety, certainty, and focus. Schüll (2012) wonderfully shows that addicted agents seek out this state in electronic gambling machines, and the reliable production of “the zone” plays an important role in making EGMs addictive.

But, importantly, I only mean to claim that *reward* uncertainty is an AMS.^17^ Still, such a restriction cries out for a clarification of what “reward” is. This is a tall order; definitions vary widely in neuroscience and cognitive science, at least partly because of the differing surrounding systems and theoretical architectures involved (Berridge & Kringelbach, 2008, p. 474; Haas, 2022; Sripada, 2025).

Still, I think a functionalist account can flesh out “reward” enough for our purposes. On this view, reward is the *main entity that grounds and is a metric for motivation*. And its processing is characterized by that set of cognitive and neural processes that I earlier referred to as the mind-brain’s internal reward system.

And so, the kind of reliability that produces flow states in gambling is not opposites with *reward* uncertainty, but rather with a kind of *affective* uncertainty picking out mood or feelings. In other words, to claim that flow states in gambling constitute a counterexample to reward uncertainty being an AMS is to mistake *affective* scaffolds for *motivational* ones.

With that distinction in hand, let’s discuss why reward uncertainty is an AMS. To give us preliminary evidence for this claim, let’s examine how reward uncertainty is found and used for addictive purposes in the two paradigmatic addictive activities we’ve been discussing: gambling and gaming.

Starting with gambling, one reason why electronic gambling machines (EGMs) are more addictive than regular gambling games (e.g., card games played around tables) is because the rules, “inner mechanisms and odds of gambling devices have always been concealed in a box” (Schüll, 2012, p. 78). Gambling machines are addictive, in other words, partially in virtue of the “purposive obfuscation” of their odds and rules (Schüll, 2012, p. 78). The shift from explicit rules and odds (such as dice rolling or deck shuffling) to implicit rules and odds (such as computer programming) is largely to blame here (Schüll, 2012, p. 78). EGMs distinguished themselves from their non-electronic counterparts “by adding an element of chance […] consumers could not be certain ahead of time how much, if anything, the machine might return. This formula proved immensely successful for gambling purveyors.” (Schüll, 2012, p. 80). In other words, EGMs made rewards more *uncertain* for the user/agent. Addictive forms of gambling possess reward uncertainty.

Moving to gaming, MMORPGs (massive multiplayer online role-playing games) are one of the most addictive kinds of video game (Berle et al., 2015; Eichenbaum et al., 2015; Flayelle et al., 2023, p. 138). And Griffiths and Nuyens (2017) observe that a “game mechanic included in all MMORPGs is the reward system involving the use of operant conditioning that leads to repetitive play because the player cannot predict when the next reward (e.g., leveling up, money, valuable in-game items) will be provided within-game” (Griffiths & Nuyens, 2017, p. 279).

But, one might object, much of a MMORPG like WoW does not involve reward uncertainty. For instance, when completing quests, one might already know the reward or not care (since many WoW players have the maximum level of experience and gear).^18^ So, how can we know that reward uncertainty is doing some work in making MMORPGs addictive?

So, to show that reward uncertainty is an AMS, I’ll point to a type of game that is strongly motivating and in which reward uncertainty plays an outsized role: Gacha games. In Gacha games, people spend actual or in-game currency for a chance at winning characters or items for their characters. Sometimes, these games have other mechanics surrounding them (e.g., battling trading cards/characters), but the essential property is reward uncertainty. Crucial for our purposes: These games are strangely addictive (C. Chen & Fang, 2023; Woods, 2024).

And so, by isolating reward uncertainty from other factors (e.g., “questing” cases in WoW), we can show that reward uncertainty in particular is an addictive motivational scaffold.

How reward uncertainty usually appears in games is through a variable ratio schedule for rewards. Variable ratio schedule delivery of reward basically means changing when you deliver rewards from a fixed time period (e.g., after 8 events) to a variable one (e.g., after 6 events, then after 10, then after 2, etc.). So, a variable ratio schedule (as opposed to a fixed one) increases the reward uncertainty. Both gambling and addictive gaming have variable ratio schedules for rewards.

Much of gambling involves a variable ratio schedule — for instance, slot machines don’t give out a jackpot or a win after a fixed number of times.

Addictive video games also have variable ratio schedules. For instance, a player might achieve “a desired reward (for example, a rare item) after repeating an in-game action (for example, killing an enemy) a variable and unpredictable number of times” (Flayelle et al., 2023, pp. 138, 141). Loot boxes – items that give undetermined in-game rewards – are also a staple of many video games and a classic example of reward uncertainty (Drummond & Sauer, 2018). When monetized (e.g., when you have to use real-world currency to buy loot boxes), loot boxes bear an uncanny resemblance to gambling (Garea et al., 2021; Hing et al., 2022; Zendle et al., 2020).

Psychological studies that examine fixed vs. variable ratio schedules in reinforcement learning provide evidence for reward uncertainty being an AMS. Variable ratio schedules increase the kinds of impulsive behavior we associate with addiction. Zeeb et al. (2017) found that “animals that had undergone unpredictable reward delivery (VR) later displayed significantly more risky decision making” than those that had experienced a fixed ratio schedule (Zack et al., 2020, p. 4; Zeeb et al., 2017). And Mascia et al. (2019) found that animals that were given amphetamines on a variable ratio schedule as opposed to a fixed one showed more risky behavior and sought out the drug more, which is evidence that variable ratio schedules can contribute to a behavioral addiction. That gives us evidence that reward uncertainty is an AMS.

More generally, reward uncertainty has been found to correlate with increased motivation to seek out a reward (Anselme, 2015).

The correlation between reward uncertainty in a reward architecture and addictiveness can plausibly be explained by the neuroscientific concept of *incentive hope*. Let me explain.

*Incentive salience* roughly refers to how much a given stimulus stands out in our subconscious, reward-based representation of the world (Berridge, 2007; Parkhurst et al., 2002; Railton, 2012, 2017). Incentive sensitization theories of addiction convincingly argue that incentive salience is overattributed to addictive stimuli. Certain neurons and neurobiological systems become *sensitized* to fire more easily and “strongly” in the presence of these stimuli, thereby increasing their motivational “pull”. Note that this “pull” shows up as a phenomenological “wanting” that is distinct from “liking” the given stimulus (Berridge, 2007, 2012, p. 1125; Smith et al., 2011; Zhang et al., 2009).

For substance/drug addictions, incentive sensitization theory claims that the dopamine release such drugs cause plays the role of overattributing incentive salience. So, if an activity releases dopamine in a similar way, we should expect addictive tendencies on account of incentive sensitization. There’s good evidence that reward uncertainty in gambling involves dopamine release (Anselme & Robinson, 2013; Zack et al., 2020). And so given that gambling is a paradigmatic addictive activity, you might think we have reason to conclude that incentive sensitization is also at work in gambling.

But that analysis, on its own, isn’t a full explanation. Incentive sensitization theory only makes claims about *certain* rewards. And reward *uncertainty* is an essential, *sine qua non* of gambling.

To fill this theoretical gap, we can look to incentive hope. Theories of incentive hope posit that “[o]rganisms exposed to uncertainty behave as if they explicitly hoped for a reward” (Anselme & Güntürkün, 2019, p. 8). Anselme and others offer a number of interesting evolutionary explanations (Anselme & Güntürkün, 2019; Anselme & Robinson, 2013). These explanations mostly argue that being motivated to seek out an uncertain reward is evolutionary advantageous, given that persistent pursuit of a given uncertain reward was oftentimes necessary to obtain it.

Importantly, incentive hope theories do *not* claim that we seek out situations in which there is reward uncertainty. Rather, they simply claim that, given a situation in which there is reward uncertainty, we will be very motivated to seek out the reward.^19^

Incentive hope thus explains why it is that reward uncertainty is so motivating – and thus why reward uncertainty is an AMS.

Furthermore, it seems as though rewards can be uncertain in two ways. For any given event, it seems as though the reward can be uncertain with respect to *whether* it is coming and with respect to its *size*.

Until now, we’ve mainly been discussing *whether* a reward is certain. But there’s good evidence to suggest that the uncertainty of the *size* of the reward also contributes to gambling and other behavioral addictions. Fiorillo et al. (2003) found that dopamine activity is “greatest when the size of the reward [is] also most variable” (Zack et al., 2020, p. 4).

In short, empirical evidence and analysis supports the claim that reward uncertainty is an AMS.

## Short Time-Horizon to Reward

Reward uncertainty usually doesn’t work alone. Some studies have suggested that, along with the uncertainty of a given reward, “concomitant signaling of reward *proximity* (time until delivery)” may be “critical” for the features of an addiction to manifest (e.g., risky behavior and reward seeking) (Howe et al., 2013; Mikhael et al., 2022; Zack et al., 2020, p. 4).

Corroborating this, Schüll looks to EGMs (again, perhaps the most addictive form of gambling) to cash out the interdependence and mutual addictive reinforcement of reward uncertainty on the one hand and the timing of rewards on the other. She does so in terms of the addictive power of the *quick resolution of uncertainty*. Machine gambling, she writes, places the player “directly in the line of chance and mediates chance in a way that grants her a sense of resolution and certainty” (Schüll, 2012, pp. 230–231). The increased reward uncertainty from machine gambling combined with the increased event frequency present in EGMs “further shrinks the time span of uncertainty, immediately resolving the event of the bet with the quick press of a button. Its ‘rapid succession of events of anticipation and consummation,’ as the Australian gambling scholar Jennifer Borrell writes, has the effect of continually collapsing an uncertain future into the present” (Schüll, 2012, pp. 205–206). This combination of reward uncertainty and higher frequency of events in a given timespan is often called *intermittent reinforcement/reward* (Voinea et al., 2024).

Thus, the *timing* of rewards also plays an important role in making an activity addictive. Specifically, the following is usually the case: the more rewards within a given period of time, the more addictive the activity.

Call an activity that possesses a *shorter time-horizon to reward* one that affects motivational processes in the mind-brain (or, if you like, the brain’s reward system) as if a positive reward was received more often within a given timespan. I’ll now show how short time-horizon to reward is a motivational scaffold that is central to addictive forms of gambling and gaming as well as providing a causal story for this link. This, in turn, will provide evidence that short time-horizon to reward is an AMS.

Gambling — an addictive activity — usually involves a short time-horizon to reward. The time between poker hands and slot spins is small. But gambling studies shows that the most addictive gambling activities — those that most often lead to problem gambling (gambling addiction) — “involve high event frequencies and short interval between stake and payout”, such as slot machines (Calado & Griffiths, 2016, pp. 609–610; Meyer et al., 2011). A higher event frequency means a shorter time-horizon to reward (more events means more rewards in a given time period). Machine gambling and EGMs are just such kinds of gambling. They have a very high event frequency (and thus shorter time-horizon to reward), and Schüll writes that “machine gambling is distinguished by its solitary, continuous, and rapid mode of wagering” (Schüll, 2012, pp. 17–18). By contrast, consider the lottery. It’s far less addictive than EGMs, and the time-horizon for rewards is comparatively much longer. One usually has to wait days or hours before one knows the lottery results.

A number of innovations that have made EGMs more addictive have also involved shortening the time-horizon to reward. One of the most prominent was the development of *multiple paylines* on EGMs. This innovation allowed gamblers to wager on multiple combinations of symbols on the EGM’s screen (as opposed to just one, such as the central line) (Schüll, 2012, p. 120). After this innovation the game “offered over 50 percent chance of winning on at least one line per spin […] it also meant that the machines were five times more reinforcing than single-line games” (Schüll, 2012, p. 120). A similar phenomenon occurred on video poker machines, with double play and triple play condensing multiple hands into one (Schüll, 2012, pp. 129–130). In short, these innovations made gambling more addictive by ensuring the gambler would end up with more rewards in less time. The time-horizon until reward was shortened, and EGMs became more addictive as a result.

Another way in which addictive gambling and games are designed to shorten the time-horizon to reward is by instantiating losses disguised as wins (LDWs). For example, on electronic gambling machines (e.g., video slots), the possibility of placing multiple bets at once allows for one to obtain a payoff from the spin that does not cover one’s initial bet. One might bet 50 credits in multiple bets on a given spin and receive 25 back. LDWs not only “generate psychophysiological responses that are qualitatively similar to full wins”, but also “promote over-estimation of the perceived frequency of wins tested after the game” (Jensen et al., 2013; Zack et al., 2020). By disguising losses as wins, LDWs shorten the time until the agent receives something they perceive to be a reward.

As for gaming, MMORPGs possess short time-horizon to reward in spades. Loot drops are very frequent events in many of these games, coming after defeating even minor enemies. Elder Scrolls Online and WoW are both clear examples of this.

But here, one might again object: If MMORPGs possess multiple AMSs, how do we know that short time-horizon to reward is doing the motivational/addictive work? To respond to this objection, I’ll focus on a game that isolates short time-horizon to reward.

There are several popular games that fit the bill for mostly focusing on event frequency as opposed to other AMSs (quantified metrics, reward uncertainty, and physically salient features). *Smash Hit* fits the bill especially well. In this game, you constantly move forward, and your goal is to keep progressing. You have metal balls that you throw to smash glass/obstacles standing in your way. Each obstacle is roughly the same distance from the next one.

*Smash Hit* has high event frequency – one cracks a lot of glass and gets through many obstacles in a given minute. It also does *not* have reward uncertainty (at least not very much of it) – no obstacle is worth more than another, and each comes at roughly the same time. The game has nice colors, but it does not possess any especially physically salient features (e.g., flashing lights, sharp contrasts, etc.). As for quantified metrics, the game does not have any point counter.

Still, one might object, the game does have the equivalent of levels, which (if one stretches the meaning of quantified metrics) could be construed as a quantified metric. This might pose a problem, as (if this is plausible) the game would not isolate short time-horizon to reward from other AMSs completely.

*Smash Hit*, however, also has a “Zen mode”. As the name implies, the Zen mode version of *Smash Hit* is less intense than the regular version. The main difference is that this version of the game has no levels, and so surely no quantified metrics while playing. It’s an endless run in which the obstacles cannot hit you – all you have to do is smash them.

And *Smash Hit* is not alone. Other games like endless runners also have Zen modes. This is evidence that short time-horizon to reward is an AMS. Zen modes – and Zen gaming in general – are a popular form of gaming (eddybox, 2008a, 2008b; Nordström, 2019; Rana, 2018), which suggests that it’s rather motivating.^20^ While there aren’t any statistics on the matter (as far as I can tell), it would not have made sense for developers to create a Zen mode if it was not improving their download numbers and engagement metrics, especially after any beta testing.

The causal mechanism underlying the motivational/addictive nature of short time-horizon to reward is a rather basic one. The mesolimbic dopamine pathway – the neural circuit primarily responsible for motivation – responds more strongly to *immediate* as opposed to *delayed* rewards (Kobayashi & Schultz, 2008). A shorter time-horizon to rewards makes rewards more immediate to us in prospect. Thus, the neural circuits involved in motivation respond more strongly.

Thus, short time-horizon to reward is a central feature of more addictive forms of gambling. That, in combination with the just-outlined causal mechanism, gives us good evidence that activities with short time-horizon to reward are more addictive. As such, short time-horizon to reward is an AMS.

## Physically Salient Features

Up until now, we’ve been talking about AMSs having to do with reward itself. But one AMS concerns *signifiers* of the reward. Signifiers are entities that are not the reward itself but that reliably predict it.

The extent to which signifiers of activity exhibit instantiations of *physically salient features* are an AMS.

What is physical salience? Let’s begin with salience *simpliciter*. Separate from incentive salience, salience *simpliciter* refers to a theoretical structure posited in the cognitive science of attention. Most cognitive scientific theories of attention posit that the best framework to understand our selection of what to attend to is a *priority map* in which various factors influence which stimuli gain priority for our attention (Awh et al., 2012; Todd & Manaligod, 2018). Among these factors are goal selection and selection history, but also *salience*. Salience refers to any number of features inherent to a stimulus that gives it greater “priority” in a given priority map – and thus makes the stimulus “call out” for attention.

Importantly, among these features are “properties of the stimulus display itself, rather than on the internal mental state of the observer” (roughly, *physical* features) that contribute to the salience of a given stimulus (Awh et al., 2012, p. 441). Collectively, these features are called *physical salience*. Some of the most prominent physically salient features include:

- *Contrasting color* with respect to a given background or other objects in the environment (Maljkovic & Nakayama, 1994; Nothdurft, 1993, 2000)
- *Having a different physical orientation* compared to a given background or other objects in the environment (Nothdurft, 1993, 2000)
- *Differing motion* compared to a given background or other objects in the environment (Folk et al., 1994; Nothdurft, 1993)
- *A tone over a noisy background* (Cusack & Carlyon, 2003; Kayser et al., 2005)

There are certainly more physically salient features that I have not listed, and there are complexities to all the features I listed.

To give an intuitive gloss to physical salience, think of how a flashing-colored light paired with repetitive, decently loud sounds would automatically draw one’s attention.

I’ll now argue that *physically salient features* are an AMS.

Gambling – especially electronic gambling machines – possesses many features that are physically salient. Flashing colored lights are common to analog and electronic slot machines – especially during wins and LDWs (Dixon et al., 2015). Flashing lights, in particular, have been shown to lead to more arousal and willingness to gamble on slot machines (Finlay et al., 2007). Sounds that are louder than the surrounding background noise are also present in gambling environments. The fact that casinos usually contain ambient background noise that is not especially loud probably helps in making these sounds physically salient (Ilicic & Baxter, 2021; Noseworthy & Finlay, 2009).

Addictive forms of gaming paradigmatically possess physically salient features. WoW and other MMORPGs, for example, possess strong contrasts in color and luminance. They also often possess entities with differing motion and physical orientation.

But lots of games have physically salient features. So, I’ll now focus on some games that emphasize their physically salient features at the expense of other AMSs. This is a bit more difficult, as it’s hard to imagine a game that *only* has physically salient features. But there are some motivating games that possess this feature in spades compared to other AMSs or features. For instance, games with strong visuals that instantiate strong luminance and color contrasts are often what I’ll call more *artistic* games like *Journey* and *Flower*.

*Journey* primarily focuses on open-world exploration and *Flower* on the journey of a collection of flowers through various environments. To the extent that there are any rewarding events (i.e., if you construe gaining a flower petal or jumping on an especially high platform as a rewarding event), both have a low frequency of them (and so longer time-horizon to reward). There is also little reward uncertainty in these games; to the extent that we can construe certain events in them as rewarding, we know the size of the reward (e.g., adding a flower petal) and we have some advanced warning as to when it is coming (e.g., you see flower petals that you can “pick up” coming in advance). There are no significant scores or quantified metrics in these games.

Both of these games, however, possess strong visuals and audio with physically salient features that capture attention. They have many different, sharply contrasting colors; varying luminances on the same screen; and varying sounds and tones.

Crucially, both of these games are played by many people. That may be partly in virtue of the rave reviews they got and the fact that they are emotional games (Clements, 2012; Dyer, 2013), but it seems to be a good explanation that they are so popular at least partly in virtue of their being substantially *motivating*. Of course, we would not expect them to be addictive – they only possess one AMS after all. But if they are at least somewhat motivating, it’s some suggestive evidence that physically salient features are an AMS.

So, addictive gambling and many forms of gaming possess physically salient features. What’s the causal explanation for this leading to addictive behavior? I see three plausible (non-exclusive) avenues.

First, Finlay et al. (2007) support the idea that the *illusion of control* is related to the relationship between flashing lights and motivation to gamble. Congruence of flashing lights with wins or LDWs, the logic goes, gives gamblers the illusion of control, leading to more arousal. From here, one could extrapolate that a false sense of security provided by the illusion of control might lead one to lower one’s cognitive defenses and thus make one’s self-control more unreliable, in turn causing more addictive behavior. Such a claim would be supported by cognitive distortion-based models of addiction, such as that proposed by Sripada (2022).

Second, physical salience could have a more *direct connection to neural pathways involving motivation*. One of the main regions in the mesolimbic dopamine pathway is the ventral tegmental area (VTA). Per incentive sensitization theory, we would expect dopamine neurons to activate in the presence of stimuli with incentive salience. But it turns out that the dopamine-neuron-heavy VTA and the structures it projects to also activate in response to physically salient stimuli (Schultz, 2010; Shao et al., 2013, p. 7). So, the VTA and the structures it projects to encode physical as well we incentive salience. Furthermore, at least one fMRI study has also suggested that the striatum (which is heavily connected to the VTA in the mesolimbic dopamine pathway) plays a role in processing salient events *simpliciter* (Zink et al., 2003). So, the striatum seems to play a role in processing physically salient as well as incentively salient stimuli. This overlap in the processing of incentive salience and physical salience suggests the plausibility of a more direct neural connection between physical salience and motivation (Schultz, 2010, p. 6).

Third, physical salience can plausibly play an important role in *maintaining our attention*. Beyond capturing our attention generally, gambling captures our attention in a specific way. Kim et al. examine how attentional biases differ between those addicted to gambling and those who are not and find that attentional bias in gambling disorder “is defined by sustained attention to gambling-related stimuli rather than faster attentional capture” (Kim, Sears, et al., 2021, p. 970). In other words, the way gambling-related stimuli capture our attention in an addictive way has to do with maintaining our attention for longer more so than capturing it more quickly at first.

Physical salience leads to attentional biases. The more an activity possesses physically salient features, the more difficult it is to shift one’s attention away from it.^21^ This allows the activity to continue to capture our attention.

From here, recall that attentional bias toward addiction-related “cues plays a role in development and maintenance of addictive behaviors” (Field et al., 2014; A. Jones et al., 2021, p. 413; Kim, Sears, et al., 2021, p. 970; Robbins & Ehrman, 2004).

So, the physically salient features plausibly allow the activity to capture our attention, making activities with physically salient features more addictive.^22^

I am agnostic as to whether one or all of these three plausible explanations of the connection between physically salient features to addiction is right. But jointly – along with the fact that activities like gambling and addictive gaming possess physically salient features – they provide good evidence that *physically salient features* is an AMS.^23^

## Addictive Motivational Scaffolding, Revisited

So, we have an account of addictive motivational scaffolding (AMSs). Namely, the following:

1. Quantified metrics
2. Reward uncertainty
3. Short time-horizon to reward
4. Physically salient features

From my analysis, it’s clear that (2) and (3) work especially effectively together.

The main claim of this paper is as follows: each of these AMSs is likely to be a difference maker in that it makes an activity more addictive.

To remind the reader of a few caveats: There are very probably more addictive motivational scaffolds than those I have identified.^24^ All the AMSs are also graded/dimensional, and so one activity that possesses one AMS in abundance might be more addictive on the whole than another with a more moderate amount of multiple AMSs. Finally, none of what I say in this paper licenses me to make strong modal claims – I don’t claim that AMSs are necessarily difference makers, for instance. There could be interaction effects I’m not taking into account.

All that being said, here are some fruits of the paper so far. First, just identifying AMSs alone offers opportunity for intervention should we want to reduce the addictiveness of activities.

Second, it’s clear that, together and if they are strongly instantiated, these four addictive motivational scaffolds are very likely to make an activity highly addictive. Note that, despite the fact that I provide plausible causal explanations for why each AMS makes an activity more addictive, I can’t make the further claim that AMSs are *jointly sufficient* for a highly addictive activity. Again, it’s plausible that there might be interaction effects for which I don’t account. But the four AMSs together and strongly instantiated make it very likely that an activity will be highly addictive. This provides a kind of explanation for the addictive nature of lots of (if not all) activities that fall under the category of behavioral addictions.

And so, we end up with an account of behavioral addiction. Not a full account, of course – we’re merely explicating one lens on the phenomenon from the perspective of 4E cognition. But it is an elucidating one.

What can we do with this account? Most prominently, we can apply it to the new seemingly addictive activities mentioned at the start of this paper (e.g., social media, pornography, digital products) in order to elucidate why they are addictive.

## Addictive Motivational Scaffolds, Social Media, and Structure

Among the new kinds of behavioral addiction becoming more salient in the Information Age, social media is especially prominent. To demonstrate the fruitfulness of my account of addictive motivational scaffolds, I’ll apply it to social media to systematically elucidate the more *structurally* addictive aspects of social media platforms.

What many literatures miss is a systematic focus on the more *structural* aspects of social media platforms that make them addictive. By *structural*, I mean everything that doesn’t have to do with the content of social media platforms and posts.

Of course, this distinction is a rough one. For one, content shapes structure (e.g., posts of different lengths appear with differing visuals on some social media platforms). But there are certainly some features of social media platforms that are more paradigmatically structural (e.g., user interface) and others that are more content-based (e.g., what kinds of topics a recommendation algorithm is pushing). And some features fall in between.

It is certainly possible to identify mental scaffolding that primarily has to do with content. But the ability of mental scaffolding to focus on external *structure* facilitates clearer theoretical insight into the more structural aspects of social media that other theoretical lenses can fail to systematize or unify.

In the vein of more structural analyses of social media, several papers give accounts of the various kinds of mental scaffolding that social media has come to instantiate. Some discuss how social media platforms’ affective scaffolding emotionally regulates us in various ways (Krueger & Osler, 2019; Steinert et al., 2022). Others touch on this idea without mentioning the word “scaffolding”. For instance, many authors have talked about how the innovation of infinite scroll on social media reduces friction in the user experience and allows users to remain “in the zone” (i.e., in a flow state) (Flayelle et al., 2023, p. 140; Montag et al., 2019; Narayanan, 2022). Voinea et al. (2024) focus on how social media is a form of attentional scaffolding.

These kinds of analyses, however, have not been applied to the addictiveness of social media through an analysis of its motivational scaffolding. By analyzing social media platforms in terms of addictive motivational scaffolds, we can elucidate how its more structural aspects create the addictive nature of social media use in a systematic and unified way. And we can identify more systematic practical interventions to reduce its addictiveness.

Furthermore, identifying AMSs in social media will also allow us to unify seemingly disparate addictive phenomena – for instance, (1) social media and (2) gambling/gaming.^25^

Here, one might make an objection along the following lines: Is it really possible for the structure/form of social media to be addictive independent of content? Social media is primarily motivating, one might think, because it involves *content* that is rewarding (e.g., sexy people on Instagram, or valuable information for our areas of interest, etc.).^26^

My reply: A lot of the content on social media seems very rewarding (indeed, sexy people doing sexy things, valuable information, etc.). But a lot of it isn’t – think random TikTok videos about bad food or AI-generated slop. Furthermore, a lot of the content seems actively harmful to our interests – the widespread phenomenon of “doomscrolling” I think demonstrates this well (B. X. Chen, 2020). We often seek out social media *despite* its content. Why? It seems as though the structure/form of the media is a natural place to look for an answer.

One might then object: Isn’t such an explanation for the addictiveness of social media focusing only on structure incomplete? I agree that a more complete answer to the question of why social media is addictive would also include content-based considerations, as well as interactions between structure and content. But the answers given up until now focus on content at the cost of structure, and so I think it important to propose a more structural account here. Additionally, no theoretical lens on addiction can be completely comprehensive, so I don’t take this kind of objection to be too worrisome.

In identifying how social media use possesses AMSs, we need to distinguish between the activities of posting and consuming social media. The vast majority of social media content is produced by a small fraction of the individuals and organizations on the platform(s). While some users exhibit behaviors consistent with addiction in their posting social media content (think of your relative or friend who cannot stop posting on a social media platform for the life of them), most users just browse social media content (J. M. Jones, 2023). This indicates that there’s an important difference between *posting* on social media and *consuming* social media. Let’s consider these separate activities, for the purposes of our analysis.

## Posting on Social Media

Social media platforms possess quantified metrics. They’re rife with icons that are meant to express some sort of pro-attitude with respect to content — likes, hearts, and upvotes are prime examples. Both Nguyen (2021) and Browning and Adams (2023) discuss how these pro-attitude icons are important for us motivationally when engaging with social media. They are often (perhaps with a view count) the only ways in which we can identify how “well” one’s post is doing.

These metrics are almost always *quantifiable*. Why? Because quantifiable metrics are necessary for large institutions. Recall the portability theory of information from STS. Drawing on the work of Theodore Porter, Nguyen (2021, p. 423) explains:

[I]nstitutional quantification is driven by an interest in making information highly portable. Rich, nuanced qualitative information is difficult to manage from any sort of informational center. We need to strip out the context-sensitive details and nuance in order to transmit it easily between contexts (Porter, 1996). This is why such quantification is beloved of centralized bureaucracies, which need to pass information to distant managers, and up many levels in the hierarchy of administration (Scott, 1998).

Social media platforms are themselves large and centralized institutions — a product of the reaggregation of data and content that characterized Web 2.0 (Napoli, 2019). Social media platforms have strong incentives to aggregate as much data as possible in order to optimize their own operations — and ultimately their profits. A precondition for this optimization is using metrics that are quantifiable.

So, posting social media involves and (barring a radical change in political economy) will continue to involve quantified metrics – an AMS.

Moving to another AMS, the rewards gained from posting on social media are also *uncertain* — both in size and whether they will occur. When posting content on social media, it’s always uncertain how much engagement a post will get. There’s not only always an uncertain chance of virality, but there’s also always uncertainty in how much engagement any given post will get in comparison to one’s previous posts and other comparable users’ (e.g., friends’) posts.

So, posting on social media involves both the AMSs of quantified metrics and reward uncertainty.

## Consuming Social Media

Consuming social media is increasingly characterized by shorter time-horizon to reward – an AMS. There are two reasons to believe this. Firstly, the general rise of short-form content on social media corroborates this. Paradigmatic examples of this trend include Instagram reels, TikTok videos, and YouTube shorts.

Secondly, recall that multiline video slot machines shortened time-horizon to reward by increasing the amount of reward in a given time period, making them more addictive. The same occurred with double play and triple play video poker machines.

There is a similar development with respect to social media. An increasing percentage of TikToks, Instagram reels, and other short-form video content are now split screen — the top half of the device plays one video and the other half of the device plays another. Usually, one of these videos (typically the bottom half) is one without sound, such as a playthrough of a given mobile game (e.g., Subway Surfer), and the other has audio. Alternatively, the device need not be split into two for there to be two forms of media at once. Another recent development in social media has been video formats in which content (e.g., a scary Reddit post or one that constitutes moral outrage porn (Nguyen & Williams, 2020)) is communicated to the user via audio and words that appear on their device while another form of content runs in the background (e.g. an attempt at a Minecraft obstacle course).

Moving onto another AMS, consuming social media also involves physically salient features. Pro-attitude icons are prominently placed throughout the user interface of social media platforms — directly adjacent to each post, in most cases. They also usually involve color contrast, luminance contrast, and/or motion contrast when one engages with the pro-attitude icon.

The activities of posting and consuming social media, then, contain the AMSs identified in this paper: quantified metrics, reward uncertainty, short time-horizon to reward, and physically salient features. The identification of these AMSs provides us with an elucidation of the more structural side of why social media use is addictive.

## Upshots and Conclusion

I’ve given an account of behavioral addiction in terms of addictive motivational scaffolds. I argued that (1) *quantified metrics*, (2) *reward uncertainty*, (3) *short time-horizon to reward*, and (4) *physically salient features* are all AMSs. Each of these AMSs is likely to be a difference maker in that it will make an activity more addictive. Together, these four AMSs, when sufficiently strong, create a “perfect storm” that is very likely to make an activity very addictive.

I have not identified all the addictive motivational scaffolds. But using the ones I did identify, I was able to elucidate the more structurally addictive side of social media, demonstrating the account’s fruitfulness.

There are two main theoretical upshots of this account. The first is that it provides a novel kind of explanation of behavioral addiction in virtue of its being at a new level of analysis – that of motivational scaffolding. This level of analysis can’t explain everything about behavioral addiction – no level can. But it is certainly a new theoretical lens on the phenomenon.

The second is that the account can unify disparate phenomena that are seemingly addictive – like gambling, gaming, and social media.

Furthermore, an important practical fruit of my analysis is that it can give us new levers for intervention in addiction that have not been fully appreciated. For instance, contingency management is a therapeutic intervention that essentially aims to change motivational scaffolding of activities that constitute or lead to using. It is promisingly effective, but chronically underused (Goodnough, 2020; Petry, 2011). The hope is that AMSs can help us refine contingency management strategies and develop other similar therapeutic practices.

AMSs also facilitate public policy interventions. To make posting and consuming social media less addictive, for instance, my analysis is informative. First, we could remove or mitigate the simplicity and/or physical salience of social media’s reward system (perhaps by limiting or removing pro-attitude icons in some manner). Or we could increase the time-horizon to reward (perhaps by delaying when pro-attitude icon counts are viewable and/or increasing time between each piece of content). Much more can be said about the policy implications of AMSs, but I leave this to future work. But such informed policy needs an empirically plausible and philosophically informative analysis of how behaviors such as social media use have become so addictive: an analysis that this article has sought to provide.

## Footnotes

1 Importantly, though papers often touch on both topics, I’m not concerned with the categorization question for the purposes of this paper.

2 In behavioral addictions, the development and maintenance of the addiction is fully exhausted by the activity itself. This is in contrast to substance addictions, where substances play the most prominent role.

3 Though, notably, I worry that Lavallee and Osler generalize their claims to behavioral addiction far too quickly, given that the empirical jury is decidedly out on whether addiction substitution occurs with respect to behavioral addictions – a claim that they seemingly rely on to make the generalization (Kim, Hodgins, et al., 2021).

4 As a caveat, I am relying on empirical evidence to make *generalizable* claims about AMSs; I am putting individual differences and vulnerabilities to the side.

5 If you’re an extended (as opposed to scaffolded) mind person, you can adjust the claims in this paper to be about processes that operate via structures that have traditionally been conceived of as external to it (but are in fact not).

6 Where sweets are placed in a supermarket might also be an example of cognitive scaffolding, since it’s reducing the cognitive demands of a task (seeking out sweets). It might also be an example of attentional scaffolding, since the sweets are probably placed somewhere that will naturally grab one’s attention (Gul et al., 2023; Voinea et al., 2024, p. 687). Similar external structures (and perhaps even identical ones) can support multiple kinds of mental scaffolding.

7 There are at least three kinds of motivational scaffolding. The first is the result of intentional human design to affect our reward systems – think of video games, vending machines, slot machines, social media, the lottery, digital menus (with, e.g., pictures of food), supermarket layout, and more. The second may bear the markers of human design but are clearly not *intentionally* designed to affect our reward system in a particular way – think of how the hierarchical structure of a corporation motivates workers, or how the timing of pay day affects our motivation on specific days. The third kind might not even bear the markers of human design. Think of how a series of rapids while you’re whitewater rafting might instigate your reward system so as to motivate you to want to do it again.

8 I thank an anonymous reviewer for pressing me on this point.

9 Motivational scaffolding can also be heavily related to forms of attentional scaffolding. If one adopts a pluralistic conception of attentional scaffolding to facilitate “richer and more productive analyses”, then it’s plausible various forms of motivational scaffolding will count as attentional scaffolding as well (Voinea et al., 2024, p. 686).

10 I also think that Timms and Spurrett (2023) are right to say that scaffolding can be *hostile* – it undermines the agent’s interests (Spurrett, 2024; Timms & Spurrett, 2023). Contrast this with *benign* scaffolding, which does not. But this distinction won’t be relevant for our purposes. I don’t make it because I want to be agnostic about whether a given addiction is in the agent’s interests or not. Such normative questions are important and are certainly relevant for defining addiction, but they’re outside the scope of this paper. I’ll specify a working conception (and its focus on the behavioral outputs of addiction) later on. But I want to emphasize that this is merely a *working* conception that aims to roughly capture the phenomenon I am concerned with in this paper; I am also agnostic about what the definition of addiction is or should be.

11 For instance, the fact that what I term “reward uncertainty” is addictive is a well-documented finding in various literatures. Flayelle et al. (2023, p. 143) also discuss at least two of the AMSs that I discuss in this paper – see especially Table 2. I expand on these (usually brief) discussions by giving more detailed causal stories as to how they affect motivational processes within the mind-brain and providing new kinds of evidence for the AMSs (by comparison to addictive gambling and gaming). I also make a unique theoretical contribution by unifying the AMSs in the theoretical framework of motivational scaffolding. The AMSs I discuss are rarely unified.

12 A brief metaphysical note: Technically, particular external structures *instantiate* motivational scaffolding (and do not, for instance, *possess* motivational scaffolding on their own) because motivational scaffolding is a *relational* property of a given object or activity in which at least two of the relata are (1) the given agent and (2) the external structure (much like affordances and mental scaffolding *simpliciter*) (Lavallee & Osler, 2024, p. 9; Timms & Spurrett, 2023, p. 57). For the sake of clarity, I use both “possess” and “instantiate”, but the latter is technically correct. I thank an anonymous reviewer for pressing me on the metaphysical nature of motivational scaffolding.

13 I am loosely drawing on the DSM-5 for the signs and symptoms in question, but I do not necessarily subscribe to their categorization of addiction or their methodology (American Psychological Association, 2013, pp. 483–484).

14 As a result, it might also be right to say that quantified metrics are an *attentional* scaffold. But I remain agonistic with respect to this claim.

15 The addiction Stroop effect’s robustness is good evidence for this fact (A. Jones et al., 2021, p. 413).

16 There exist strong disputes about the role of dopamine in reward learning, which in turn has led to a split between “learning dysfunction” and “incentive sensitization” theories of addiction. Despite this theoretical split, I take it that both sides would agree that various kinds of learning – e.g., habit learning – play an important role in the development and maintenance of an addiction (whether direct or indirect).

17 I am thankful to an anonymous reviewer for pressing me on this point.

18 I’m indebted to an anonymous reviewer for pressing me on this point.

19 Here, some might ask how we explain people seeking out addictive activities (gambling, gaming, etc.) in the first place. I’d first point to other AMSs as possible explanations. But it’s also the case that some populations might be more vulnerable to seeking out addictive activities in the first place. Particularly strong candidates for vulnerability might be those in the grips of mechanisms that are common to theories of the ‘dark side’ of addiction (i.e., opponent process theories of addiction), such as stress and anxiety circuits (Koob, 2017; Lembke, 2021a).

20 Zen Modes might not be as popular as the main game, but that’s just what we’d expect, given that they have fewer AMSs.

21 There is a growing literature concerning the time course of physical salience – that is, how long the heightened salience lasts. In particular, there’s some evidence to suggest that the attention-grabbing effects of physically salient features do not last all that long (Dombrowe et al., 2010; Donk & van Zoest, 2008; Krüger & Scharlau, 2022). This could potentially undermine the claim that physical salience plays an important role in *maintaining* our attention. I think this thought is misguided. Even if salience effects don’t last for that long, they can still *significantly increase* the amount of time people attend to a given object. This, in turn, would allow physical salience to play an important role in maintaining attention on addictive stimuli.

22 If this is the correct causal mechanism, then physically salient features would plausibly be an AMS by functioning as an attentional scaffold.

23 An anonymous reviewer raises an objection along the following lines: lots of seemingly non-addictive entities possess physically salient features (e.g., advertisements, traffic signs, etc.). Notice that this fact is still compatible with the main claim of this paper: that AMSs are *likely to be difference-makers in making an activity more addictive*. This weakness of this claim also restricts me from making modal claims like “these four AMSs are *jointly sufficient* for an activity to be more addictive” – there could be interaction effects I’m not accounting for, among other things. But even so, the force of the objection can be captured with this question: How can a *traffic sign* be addictive in any sense? That just seems wrong! My reply is as follows. One thing to note here is that each of the AMSs are graded/dimensional. A traffic sign is not nearly as physically salient as a strobe light or jackpot lights and sounds. So, if there’s a sense in which physical salience is directly addictive, then a traffic sign won’t be nearly as motivating as jackpot lights and sounds, even if it’s somewhat motivating. Still, it might seem weird to say that there’s such a “direct” connection between physical salience and motivation. But note that, on my account, it’s perfectly consistent to say that physical salience might not be directly motivating, but rather that it a more facilitative role. Perhaps there’s an elective affinity between physical salience and a factor that is more directly motivating – for instance, the illusion of control. It could also be that physically salient features often appear in contexts where it’s used to maintain attention in such a way that is addictive. Or, more strongly, physically salient features could just be causal contributors to a larger process that is more directly motivating/addictive. If any of these were the case, it would still be true that physical salience “is likely to be a difference maker in that, if it is instantiated, it is likely to make an activity more addictive than it otherwise would have been.” And so it’s consistent with my account. Being more sanguine about physical salience’s role in motivation, though, would push against causal explanation (2) of the physical salience section – i.e., a direct neural connection between motivation and physical salience. But given that (2) has some good empirical evidence in favor of it, I don’t want to rule it out as a possibility. I want to make room for the weird view, as I don’t want to reject empirical evidence just because it seems unintuitive. So, I maintain my agnosticism between causal explanations.

24 For instance, social reward (e.g., positive reinforcement from peers) probably plays an important role in many behavioral addictions (Voinea et al., 2024). They also very probably play a role in the most addictive kinds of gaming like MMORPGs, given their inherently social nature. And there’s good arguments from other kinds of addiction (e.g., substance addiction) that the communities one forms can play an important role in maintaining the addiction (Lavallee & Osler, 2024). So, social reward could be an addictive motivational scaffold – or at least certain instantiations of it. But ultimately, I do not include this as an addictive motivational scaffold in this paper because the AMSs I focus on have important upshots for an underdiscussed aspect of behavioral addictions – namely more *structural* features in virtue of which activities are addictive. I’m indebted to an anonymous reviewer for inspiring me to make this clarification.

25 Importantly, in this paper, I am not interested in the debate concerning whether there is or ought to be a category called “social media addiction” (Carbonell & Panova, 2017; Sun & Zhang, 2021). This is importantly different from an analysis of the addictiveness of social media.

26 I’m indebted to an anonymous reviewer for raising this objection.
